# Generation of Monoclonal Antibodies against Variable Epitopes of the M Protein of Rabies Virus

**DOI:** 10.3390/v11040375

**Published:** 2019-04-23

**Authors:** Jie Liu, Wen Zhao, Wanting He, Ningning Wang, Jingyin Su, Senlin Ji, Jian Chen, Dong Wang, Jiyong Zhou, Shuo Su

**Affiliations:** 1 MOE Joint International Research Laboratory of Animal Health and Food Safety, Jiangsu Engineering Laboratory of Animal Immunology, Institute of Immunology and College of Veterinary Medicine, Nanjing Agricultural University, Nanjing 210095, China; 2016107063@njau.edu.cn (J.L.); 2017207031@njau.edu.cn (W.Z.); vet_he@163.com (W.H.); 17416104@njau.edu.cn (N.W.); xiao_anny@yeah.net (J.S.); senlinji@163.com (S.J.); 2 China Institute of Veterinary Drug Control, Beijing 100081, China; chererdong@126.com (J.C.); wangdong420@126.com (D.W.); 3 Key laboratory of Animal Virology of Ministry of Agriculture, Zhejiang University, Hangzhou 310058, China; jyzhou@zju.edu.cn

**Keywords:** rabies virus, monoclonal antibody, matrix protein, epitope, antigenic difference

## Abstract

Rabies virus (RABV), the causative agent of rabies, is highly neurovirulent for warm-blooded animals with a mortality rate of up to 100%. The RABV matrix protein (M) is required for virus particle assembly and budding. However, little is known about antigenic differences in the M protein. In this study, five monoclonal antibodies (mAbs), designated 3B9, 4A1, 2B11, 2C1, and 4B11, against the RABV M protein were generated using a recombinant M protein. All five mAbs reacted with the CVS-11 strain but showed no reactivity against the HEP-Flury strain in indirect immunofluorescence and western blotting. The epitope targeted by these mAbs was further identified by peptide scanning using GST-fused peptides. The ^25^PPYDDD^30^ peptide was defined as the minimal linear epitope. Alignment of amino acid sequences and phylogenetic analysis of different RABV strains indicated that the variable epitope ^25^PPDGDD^30^ is only present in the HEP-Flury and variant Flury strains of clade III, while the other strains resembling ERA and SRVA9 within the clade had another variable epitope, ^25^PLDDDD^30^. A Y27D mutation within the epitope was found among the rest of the RABV strains distributed in different clades. However, a single D28G mutation eliminated the reactivity of these five mAbs. In addition, the mAbs were able to recognize wildtype RABV strain in indirect immunofluorescence and western blotting and detect RABV-infected brain tissue using immunohistochemistry. The newly established mAbs and identified epitope may facilitate future investigations in the structure and function of the M protein and the development of diagnostic methods for the detection of different RABV strains worldwide. Most importantly, the epitope recognized by the mAbs against M protein might serve as a novel target for the development of a vaccine targeting RABV virulent strains.

## 1. Introduction

Rabies, caused by the rabies virus (RABV), is an acute progressive zoonotic disease attacking the central nervous system. RABV mainly invades the human body through damaged skin or mucous membranes. Once encephalomyelitis symptoms appear, the mortality rate is almost 100% [1,2]. Although the total number of reported cases is decreasing yearly, rabies remains a permanent threat to human health [3] with more than 59,000 deaths worldwide caused by rabies each year (https://www.who.int/).

RABV belongs to the genus *Lyssavirus* of the *Rhabdoviridae* family. It contains a negative-sense and single-stranded linear RNA genome that encodes five proteins: nucleoprotein (N), phosphoprotein (P), matrix protein (M), glycoprotein (G), and RNA-dependent RNA polymerase (L) [4]. The M protein is the smallest structural protein consisting of 202 amino acids [5] and existing as two isoforms: Mα and Mβ [6]. The M protein, as a connector between the viral nucleocapsid and envelope [7], determines the budding and bullet shape of the virus [8,9]. Recombinant RABV lacking M protein are unable to be effectively packaged into the typical bullet-shaped virus particles [10]. Further, L domains of the M protein mediate virus budding through interaction with host cell proteins [11]. Moreover, Finke identified the M protein of RABV as a factor which inhibits transcription and stimulates replication [12]. The M protein of RABV significantly affects pathogenicity and virus spread probably through interacting with the G protein [13], is involved in inducing TRAIL-mediated apoptosis in RABV-infected nerve cells [14], and can interact with the mitochondria to induce cell apoptosis [15].

The genotype of RABV can be divided into three clades based on M gene [16]. However, it is unclear whether genetic variation leads to antigenic differences among different genotypes. Monoclonal antibodies (mAbs) against virus proteins, with virus neutralizing activity and high specificity to the antigen, are used not only for diagnosis and therapy, but also for distinguishing antigenicity among different strains [17]. A previous study delineated seven antigenic region groups related to the M protein using twenty-one M mAbs. In addition, the M mAb 3-9-16 was reported to recognize a linear epitope located at the N-terminus. Based on the exposure of this linear epitope, M proteins could be divided into two isoforms, Mα and Mβ [6]. Amino acid substitution at position 95 changed RABV virulence [18]. However, it is unknown that whether the amino acid substitutions lead to antigenic difference. To date, there are no reports regarding the precise epitopes of the RABV M protein. The identification and characterization of M epitopes will help us get a better understanding of antigenic variations among different RABV strains. In this study, we generated RABV-specific mAbs using a prokaryotic-expressed M protein as immunogen, and we defined the epitope targeted by these mAbs.

## 2. Materials and Methods

### 2.1. Viruses and Cells

Human neuroblastoma (SK-N-SH) cells and baby hamster kidney (BHK-21) cells were cultured in minimum Eagle’s medium (MEM) (HyClone, Logan, Utah, USA) and Dulbecco’s modified Eagle’s medium (DMEM) (HyClone, Logan, Utah, USA) supplemented with 10% fetal bovine serum (FBS) (Gibco, California, USA) at 37 °C in a 5% CO_2_ atmosphere. Challenge virus standard 11 (CVS-11) and avirulent HEP-Flury strain were preserved in our laboratory, wildtype RABV strain was generously provided by Dr. Changchun Tu and Dr. Ye Feng. These three RABV strains were preserved in our laboratory and propagated in SK-N-SH cells.

### 2.2. Expression and Purification of Recombinant M Protein

The full-length M gene (GenBank accession: GQ918139) of the RABV CVS-11 strain was amplified by reverse transcription-PCR (RT-PCR) using total RNA extracted from CVS-11-infected SK-N-SH cells as template. The primer sequences used for amplification of the M gene are shown in Table 1. PCR products were purified using the AxyPrepTM PCR Clean up Kit (Axygen, Hangzhou, China) and cloned into the expression vector pET-28a (+) between the *Hin*dIII and *Kpn*I sites. The resulting plasmid was named pET-28a-M and transformed into *E. coli* BL21 (DE3) competent cells for protein expression. After induction with 1 mM isopropyl-β-D-thiogalactopyranoside (IPTG) for 5 h at 37 °C, cells were collected, washed with phosphate-buffered saline (PBS), resuspended in PBS, and sonicated for 10 min on ice. The supernatant was discarded and the pellet was dissolved in an equivalent volume of buffer A (8 M urea, 300 mM NaCl and 50 mM Na_2_HPO_4_, pH 8.0) for 1 h at 37 °C. The fraction of dissolved pellet was analyzed using SDS-polyacrylamide gel electrophoresis (PAGE) followed by colloidal coomassie staining. The recombinant M protein was purified using NTA-agarose affinity resin (QIAGEN, Hilden, Germany) according to the manufacturer’s instructions. Finally, the purified recombinant M protein was separated using SDS-PAGE, transferred onto nitrocellulose (NC) membranes (GE Healthcare, Massachusetts, USA), and subjected to Western blotting (WB) using mouse anti-His mAb (1:1000; Abcam, Cambridge, USA) as the primary antibody and HRP-conjugated goat anti-mouse IgG as the secondary antibody (1:10,000; KPL, Gaithersburg, MD, USA). The purified protein was intended to immunize mice as antigen through concentration analysis with bicinchoninic acid (BCA) assay kit (Thermo, Waltham, MA, USA).

### 2.3. Preparation of Monoclonal Antibodies Against the M Protein

Six-week-old female BALB/c mice were injected subcutaneously with 200 μg of the purified recombinant M protein at days 0, 14, and 28. A week after the third immunization, the titre of serum antibodies of all mice was measured using ELISA as ELISA is a useful tool for determining serum antibody concentrations [19]. Three days after the last boost with 100 μg of the purified M protein, mice, with the highest serum antibody titre (1:25,600), were euthanized. Splenocytes of the mouse were collected and fused with mouse myeloma cells (SP2/0) using polyethylene glycol 2000 (Sigma, California, USA). The other spleens were fused with SP2/0 in the next time. Hybridomas were cultured in RPMI 1640 medium (HyClone, Logan, UT, USA) supplemented with hypoxanthine-aminopterin-thymidine (HAT) (Sigma, California, USA) for 14 days. Indirect ELISA coated with the purified recombinant M protein and indirect immunofluorescence assays (IFA) with RABV-infected SK-N-SH cells were used for screening positive hybridomas. The positive clones were sub-cloned by limiting dilution for three more times and further characterized using IFA. Finally, hybridomas stably producing mAbs were generated. Positive hybridomas were used to immunize the paraffine-primed BALB/c mice to produce ascites [20]. All mAbs against the RABV M protein from ascites were further identified using WB and IFA. Moreover, the isotype of the mAbs was identified using the mouse monoclonal antibody isotyping kit (Thermo, Waltham, MA, USA) according to the manufacturer’s instructions. All animal experiments and procedures involving animals were approved by the Institutional Animal Care and Use Committee of Nanjing Agricultural University, Nanjing, China (No. SYXK2017-0007, February 2017, Institutional Animal Care and Use Committee of Nanjing Agricultural University) and met the standard of the International Guiding Principles for Biomedical Research Involving Animals.

### 2.4. Screening of Anti-RABV M Antibodies Using Indirect ELISA

Recombinant M protein was used for detecting their reactivity with mAbs against the M protein using indirect ELISA. Briefly, 100 μL of recombinant M protein (2 μg/mL in buffer Na_2_CO_3_/NaHCO_3_) were added into each well of ELISA plates as the coating antigen. After 2 h at 37 °C, plates were washed three times with PBST (PBS with 0.25% Tween-20). The plates were then blocked with 5% non-fat milk in PBS for 1 h at 37 °C, washed three times, and incubated with the supernatant of hybridomas for 1 h at 37 °C. RABV-positive mouse serum and the supernatant of SP2/0 cells were used as the positive control and negative control, respectively. After washing three times, the plate was incubated with 100 μL of HRP-conjugated goat anti-mouse IgG (1:10,000; KPL, Gaithersburg, MD, USA) at 37 °C for 1 h. After three washes, 3,3′,5,5′-tetramethylbenzidine (TMB) was added and allowed to react for 10 min at 37 °C. Finally, the reaction was terminated with 50 μL of 2 M H_2_SO_4_. The optical density (OD value) at 450 nm was read using an automatic ELISA plate reader (TECAN, Männedorf, Switzerland). Results with ratios of OD_450nm_ attached to the tested samples 2.1 times higher compared to the negative control were regarded as positive [19].

### 2.5. SDS-PAGE and Western Blotting

SK-N-SH cells infected with RABV for 72 h were collected and lysed in NP-40 lysis buffer (Beyotime, Shanghai, China). Cell lysates were mixed with 4× loading buffer (Solarbio, Beijing, China) and boiled at 100 °C for 15 min. Protein samples and recombinant M protein samples were separated using SDS-PAGE with a 10% polyacrylamide gel [21] and transferred onto NC membranes (GE Healthcare, Amersham, UK). After blocking with 5% non-fat milk at 37 °C for 1 h, NC membranes were washed three times with PBST and incubated with the undiluted supernatant of hybridoma cells or mouse anti-His antibody (1:1000; Abcam, Cambridgeshire, UK) and anti-N mAb (produced in our laboratory) [22] as primary antibody at 4 °C overnight. Then, the membrane was incubated with HRP-conjugated goat anti-mouse antibody (1:10,000; KPL, Gaithersburg, MD, USA) as secondary antibody at 37 °C for 1 h. Membranes were then washed and the target protein bands were detected with enhanced chemiluminescence (ECL) (Vazyme, Nanjing, China).

### 2.6. Indirect Immunofluorescence Assay

SK-N-SH cells were grown to approximately 80% confluency and inoculated with CVS-11, HEP-Flury and wildtype RABV strain at a multiplicity of infection (MOI) of 1 for 2 h, then the virus inoculum was removed and replaced by cell maintenance medium with 2% FBS, which maintains cell growth at a lower rate and prevents overgrowth. Cells were incubated for 48 h and fixed with cold methanol-acetone (1:1) at −20 °C for 2 h. Plates were blocked with 5% non-fat milk at 37 °C for 1 h. After three washes with PBST, plates were incubated with the mAbs for 1 h at 37 °C. RABV-positive mouse serum and supernatant of SP2/0 cell were used as the positive control and negative control, respectively. Fluorescein isocyanate (FITC)-conjugated goat anti-mouse IgG (1:500; KPL, Gaithersburg, MD, USA) was added as secondary antibody at 37 °C for 1 h. After three washes with PBST, the immunofluorescent staining was observed under a fluorescent microscope (Nikon, Tokyo, Japan) [23].

### 2.7. Identification of M Protein Epitopes

To identify the precise epitopes of the RABV M protein, the M polypeptides were truncated according to the B cell epitope predication tool (https://npsa-prabi.ibcp.fr/cgi-bin/npsa_automat.pl?page=/NPSA/npsa_server.html). A series of overlapping and truncated fragments of the M gene amplified and cloned into the pET-32a (+) vector were expressed in *E. coli* BL21 (DE3) as fusion proteins containing the 6× His tag. After two rounds of truncations, the reactive polypeptide was shortened to 24–32 aa. In the final round, the 24–32 aa peptide was truncated with one by one residue from both ends, cloned into the pGEX-4T-1 vector, and expressed in *E. coli* BL21 (DE3). Primer sequences are listed in Table 2. The epitope sequence was finally determined when the smallest binding peptide recognized by the mAbs was identified. To explore the reactivity of amino acid mutations of M protein with mAbs, three epitope fragments with single mutations of P26L, Y27D, or D28G were cloned into the pGEX-4T-1 vector and expressed in *E. coli* BL21 (DE3). Complementary primers used for synthesizing mutant epitope fragments are shown in Table 3. The reactivity of truncated and mutant fragments with mAbs above was tested using WB.

### 2.8. Alignment of Epitope Sequences of the M Protein

The alignment of amino acid sequences was performed using MEGA 7.0.26 software (https://www.megasoftware.net/home, University Park, PA, USA). The variation of the epitope among different clades was defined by a ML (maximum likelihood) tree constructed with RAxML (v8.2.10) (https://cme.h-its.org/exelixis/web/software/raxml/index.html, Heidelberg, Germany) based on the GTR+G+I substitution model. All nucleotide and protein sequences were retrieved from Genbank.

### 2.9. Application of mAbs in the Detection of RABV by Immunohistochemistry

The mAbs were used to detect RABV of virus-infected mice by immunohistochemistry. Six to eight week-old male C57BL/6 mice were inoculated intracranially with the 30 μL of CVS-11 and HEP-Flury (virus titre = 1 × 10^5^ TCID_50_/mL) and euthanized 7 days later. Brain samples of CVS-11 and HEP-Flury-infected mice were collected and fixed in 10% formalin solution for at least 48 h at room temperature. Tissues were dehydrated and embedded in paraffin, cut, and sections were mounted onto glass slides and dewaxed following routine methods. Antigen retrieval was conducted by incubation with 2 mg/mL pepsin in Tris-HCl pH 2.0 at 37 °C for 10–20 min. Paraffin sections were blocked with 3% peroxide-methanol at room temperature followed by incubation with mAbs 3B9 and 4A1 at 37 °C for 2 h. RABV-positive mouse serum and supernatant of SP2/0 cells were used as the positive control and the negative control, respectively. After washing with ddH_2_O, paraffin sections were incubated with labeled polymer-HRP anti-mouse at 37 °C for 1 h. Sections were washed with PBS (pH 7.4) and incubated with DAB substrate chromogen (MAIXIN. Bio, Fuzhou, China) at room temperature without light for 10 min. Sections were counterstained with hematoxylin and Scott’s bluing solution for 1 min. Sections were analysed using optical microscopy (Nikon, Tokyo, Japan).

## 3. Results

### 3.1. Expression and Purification of the M Protein

**Figure 1:**
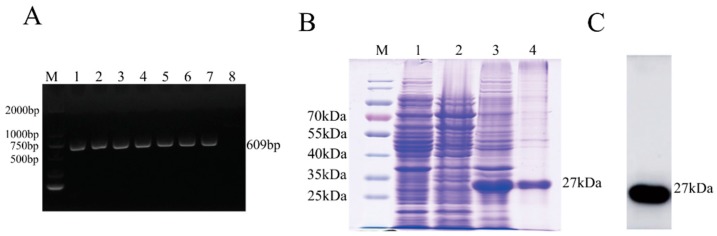
Expression of the RABV M protein. (**A**) Amplification of the M protein fragment of the CVS-11 strain. Lane M, DNA marker; lanes 1–7, full length of M gene; lane 8, negative control. (**B**) SDS-PAGE analysis of M protein expression. Lane M, protein marker; lane 1, lysates from *E. coli* BL21 (DE3) transformed with the recombinant plasmid pET-28a-M without IPTG induction; lane 2, cell supernatant after sonication from *E. coli* BL21 (DE3) transformed with pET-28a-M with IPTG induction; lane3, cellprecipitation after sonication from *E. coli* BL21 (DE3) transformed with pET-28a-M with IPTG induction; lane 4, purified his-M. (**C**) Western blotting (WB) analysis of purified his-M. Lane 1, purified His-M.

A 609 bp fragment of the M gene from the CVS-11 strain was amplified (Figure 1A), cloned into the expression vector pET-28a (+), and transformed into BL21 (DE3) cells for fusion protein expression. As shown in SDS-PAGE analysis, the M protein was expressed with a predicted molecular mass of approximately 27 kDa and mainly exist in cell precipitation after sonication (Figure 1B). WB analysis showed that the purified His-M protein could react with mouse anti-His mAbs (Figure 1C).

### 3.2. Generation and Characterization of mAbs Against the M Protein

**Figure 2:**
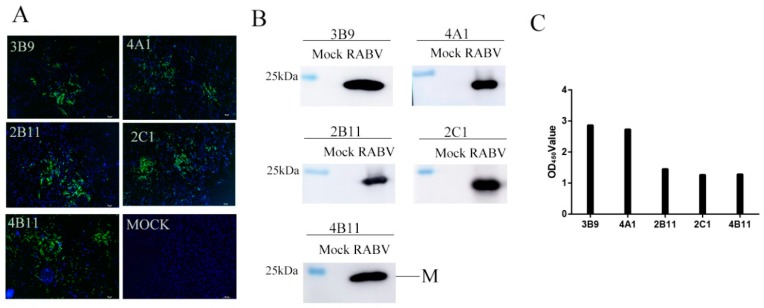
The reactivity of monoclonal antibodies (mAbs) against M protein. (**A**) Indirect immunofluorescence assays (IFA, 100×). SK-N-SH cells were infected with CVS-11 for 48 h, and then cells were fixed and incubated with monoclonal antibodies (mAbs) 3B9, 4A1, 2B11, 2C1 and 4B11. (**B**) WB analysis. Rabies virus-infected SK-N-SH cell lysates were incubated with mAbs 3B9, 4A1, 2B11, 2C1, and 4B11. (**C**) Determination of the reactivity of mAbs against recombinant M protein of CVS-11 in the indirect enzyme-linked immunosorbent assay (ELISA).

**Figure 3:**
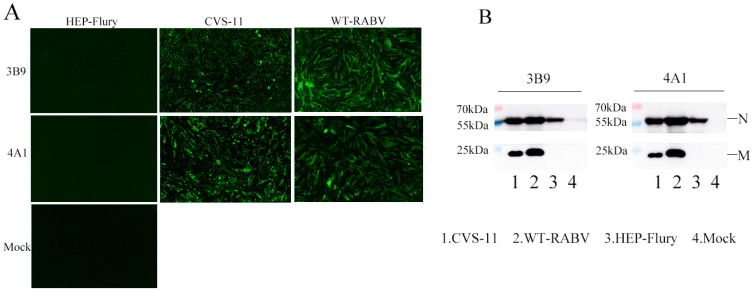
Specificity of mAbs 3B9 and 4A1 against M protein of the HEP-Flury and wildtype-RABV strains analysed using IFA and WB. (**A**) IFA (100×). BHK-21 cells were infected with CVS-11, HEP-Flury, and wildtype-RABV strain for 48 h and then incubated with mAbs 3B9 and 4A 1. (**B**) WB analysis. SK-N-SH cells were infected with CVS-11, HEP-Flury, and wildtype-RABV strain. Lysates were then incubated with anti-M mAbs 3B9, 4A1, and anti-N mAb.

After three immunizations, spleen cells of mice with the highest serum antibody titre (1:25,600) were fused with SP2/0 cells. Ten days later, supernatants secreted by hybridomas were tested using ELISA and IFA. More than 100 positive clones were selected after the first screening. After three further screens, five mAbs against the M protein were finally obtained and named 3B9, 4A1, 2B11, 2C1, and 4B11. The reactivity of the five mAbs was tested using IFA and WB. The results showed that all five mAbs reacted with the M protein of RABV-infected SK-N-SH cells in IFA (Figure 2A) and WB (Figure 2B). Isotype determination showed that 3B9, 4A1, and 4B11 mAbs were subclass IgG2bκ; 2B11 was subclass IgG2aκ; and 2C1 was subclass IgAκ. The reactivity of HEP-Flury and a wildtype RABV strain with all mAbs was examined based on IFA and WB in HEP-Flury-infected cells. However, mAbs showed reactivity with wildtype RABV strain but not with HEP-Flury (Figure 3A,B, only 3B9 and 4A1 are shown), indicating that the antigenic epitope identified by the mAbs was different in HEP-Flury and CVS-11. Then, we selected mAbs 3B9 and 4A1, which showed stronger reactivity against the M protein in indirect ELISA (Figure 2C), for further characterization.

### 3.3. Mapping of the Epitopes of M Protein

**Figure 4:**
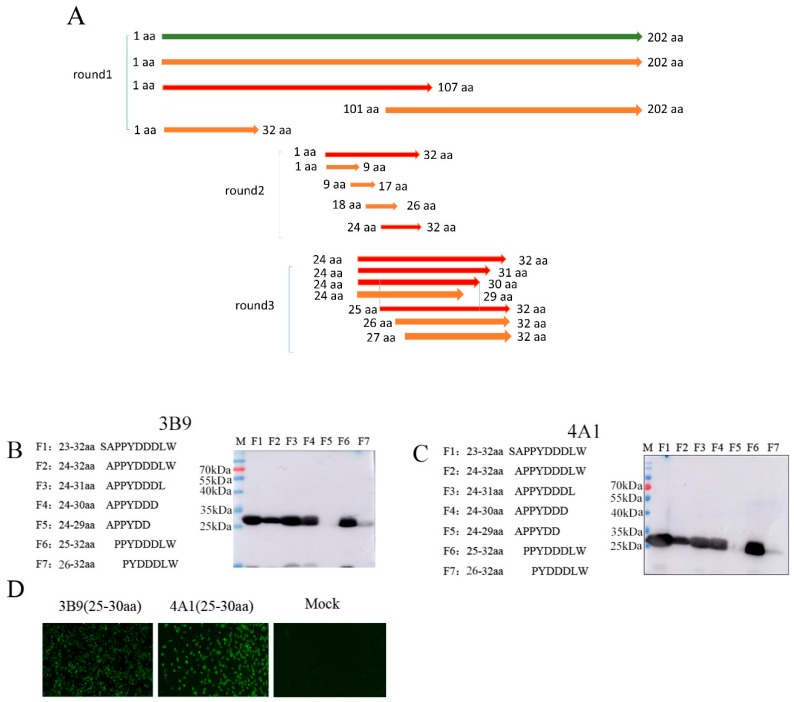
Analysis of M protein linear epitopes using WB and IFA. (**A**) Schematic representation of the RABV M protein fragments used for epitope mapping. The red fragment represents the regions that react with the corresponding mAb 3B9 and mAb 4A1. (**B**) Epitope mapping of mAb 3B9 using WB. Truncated fragments were detected with mAb 3B9, mAb 3B9 specifically reacted with proteins containing the ^25^PPYDDD^30^ sequence. F1: 23–32 aa; F2: 24–32 aa; F3: 24–31 aa; F4: 24–30 aa; F5: 24–29 aa; F6: 25–32 aa; F7: 26–32 aa. (**C**) Epitope mapping of mAb 4A1 using WB. Truncated fragments were detected with mAb 4A1, mAb 4A1 specifically reacted with proteins containing the ^25^PPYDDD^30^ sequence. F1: 23–32 aa; F2: 24–32 aa; F3: 24–31 aa; F4: 24–30 aa; F5: 24–29 aa; F6: 25–32 aa; F7: 26–32 aa. (**D**) Reactions between the mAbs and truncated M protein using IFA (100×). The truncated M protein containing 25–30 aa sequences was cloned into pCMV-Flag vector and transfected into BHK-21 cells then subjected to incubation with mAbs 3B9 and 4A1.

Epitope mapping was performed with truncated proteins. After three rounds of screening (Figure 4A), all mAbs reacted with each of the 25–32 aa, 24–30 aa, and 25–30 aa fragments (Figure 4B, Figure 4C, and Figure 6B, only 3B9 and 4A1 are shown), indicating that the epitope recognized by mAbs was the polypeptide ^25^PPYDDD^30^. To further identify the M protein antigenic epitopes, the fragment 25–30 aa was cloned into the pCMV-Flag vector and transfected into BHK-21 cells. The mAbs recognized the truncated M protein expressed in transfected BHK-21 cells using IFA (Figure 4D, only 3B9 and 4A1 are shown), suggesting that the polypeptide ^25^PPYDDD^30^ was the minimal linear epitope required for binding of mAbs 3B9 and 4A1.

### 3.4. Alignment of M Protein Epitope Sequences

**Figure 5:**
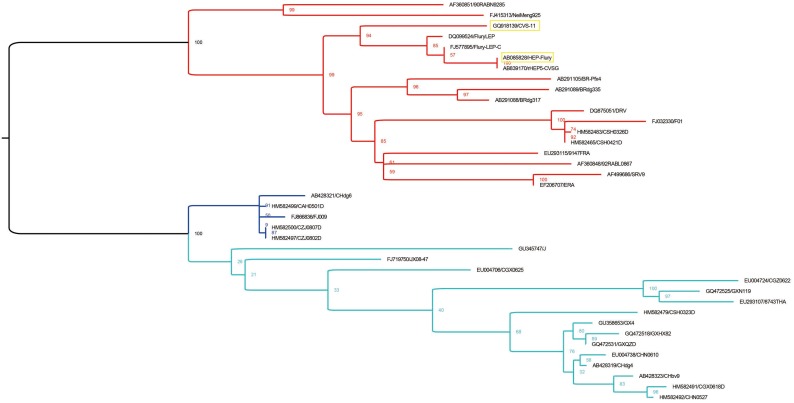
Phylogenetic tree of the M gene of RABV. The ML (maximum likelihood) tree was constructed with RAxML (v8.2.10), based on the GTR+G+I substitution model. Bootstrap values were calculated based on 1000 replicates. The tree can be divided into three clades: clade I (blue), clade II (light blue), and clade III (red). The yellow box indicates the two RABV strains used in this study.

**Figure 6:**
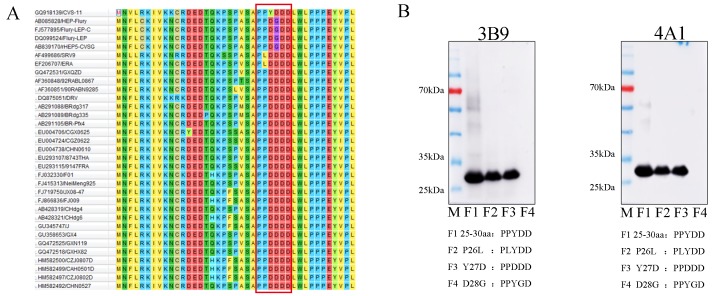
Amino acid alignment of the M protein. (**A**) Amino acid alignment of the M protein using the MEGA 7.0.26 software. The minimal epitope of (^25^PPYDDD^30^) is shown in the red box. (**B**) The reactivity of mAbs against amino acid mutations of P26L, Y27D, or D28G epitopes, mAbs 3B9 and 4A1 reacted with P26L or Y27D single mutations but not with the D28G single mutation. F1: 25–30 aa PPYDDD; F2: 25–30 aa (P26L) PLYDDD; F3: 25–30 aa (Y27D) PPYDDD; F4: 25–30 aa (D28G) PPYGDD.

According to a previous study, phylogenetic analysis of the M gene of different RABV strains revealed they were divided into three groups: clade I (blue), clade II (light blue) and clade III (red) [16] (Figure 5). The CVS-11 and vaccine strains such as ERA, SRV9, HEP-Flury, and variant Flury are within clade III (Figure 5). Alignment of amino acid sequences indicated that the variable epitope ^25^PPDGDD^30^ is only present in the HEP-Flury and variant Flury strains, while the other strains resembling ERA and SRVA9 have another variable epitope: ^25^PLDDDD^30^. Phylogenetic analysis showed these RABV strains belong to clade III, while the rest of the RABV strains distributed in different clades had the variable epitope ^25^PPDDDD^30^ (Figure 6A). In addition, we found that the single mutations P26L or Y27D do not affect the reactivity of two mAbs, but mutation D28G eliminates the binding of mAbs with mutant epitopes (Figure 6B).

### 3.5. Reactivity of mAbs with RABV in the Brain Tissue of Infected Mice Using Immunohistochemistry (IHC)

**Figure 7:**
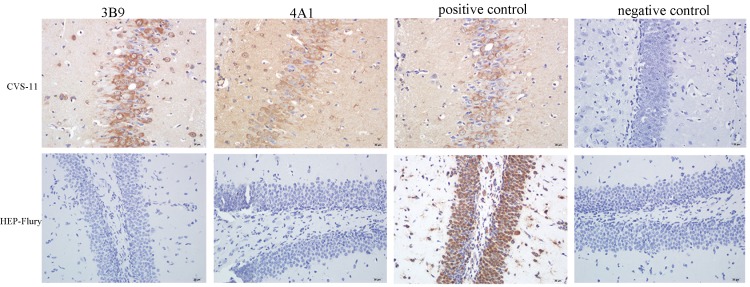
Immunohistochemical staining of RABV-infected mice brain tissue sections. Two mAbs, 3B9 and 4A1, were used for RABV detection in immunohistonchemistry (IHC, 500×). The mAbs could stain the pyramidal neurons of the cerebral cortex in brain tissues of CVS-11-infected mice but showed no reactivity with brain tissues of the HEP-Flury-infected mice.

To determine whether these two mAbs could be used for RABV detection in IHC, they were tested in brain tissues from mice experimentally infected with RABV. CVS-11-infected mice showed clinical signs including loss of body weight, decreased appetite, loss of fur lustre, and some even death, while control mice appeared normal. Two mAbs, 3B9 and 4A1, were able to specifically stain the pyramidal neurons of the cerebral cortex of the brain tissues of CVS-11-infected mice but showed no reactivity with brain tissues of HEP-Flury-infected mice (Figure 7).

## 4. Discussion

Rabies is a severe zoonotic infectious disease with nearly 100% fatality rate once clinical symptoms develop [24,25]. It has been reported worldwide and poses a threat to animal and public health. The RABV M protein participates in viral assembly and budding, it inhibits nuclear transport, regulates host transcription, and induces cell apoptosis. In addition, the M protein participates in the host immune response by interacting with the G protein [13]. Here, five mAbs against M were generated and characterized by immunizing BALB/c mice using the recombinant M protein of the RABV CVS-11 strain. All mAbs could bind to the recombinant and native M protein of RABV CVS-11 strain. However, the RABV HEP-Flury strain could not be recognized by these mAbs. Based on these results, it can be concluded that the antigenic epitopes of the CVS-11 and HEP-Flury strains are different, thus we determined the precise epitopes of M protein recognized by the mAbs.

Due to it’s high specificity to antigen, mAbs is a useful tool for mapping antigenic epitopes [19]. MAbs against RABV were first described in 1978 [26]. Since then, many mAbs against different viral proteins (P, N and G proteins) have been produced and used for epitope identification. In 2005, ^226^KLCGVL^231^ was identified as the minimal binding region by two mAbs against the G protein [27]. However, the identification of the precise epitopes of M proteins is still scant. The RABV M protein, which is encoded by 609 nt, comprises 202 amino acids with a molecular weight of 27 kDa [5]. Two antigenic regions (17–72 aa and 50–171 aa) containing linear epitopes were previously identified using mAbs against the M protein [28]. In our study, the precise epitopes recognized by the mAbs were mapped using peptide scanning, indicating ^25^PPYDDD^30^ is the minimal linear epitope. In addition, the epitope could be recognized when expressed in BHK cells using IFA. To our knowledge, this is the first record of precise epitope mapping of the RABV M protein.

Alignment of amino acid sequence showed that the precise epitope in HEP-Flury and variant Flury strains had two variable amino acids (Y27D and D28G) in relation to the CVS-11 strain. HEP-Flury and variant Flury strains belong to clade III; however, the ERA and SRV9 strains of clade III had the epitope with two variable amino acids (P26L and Y27D). The rest of the strains of clade I, clade II, and clade III shared a Y27D mutation in the epitope suggesting that ^25^PPDDDD^30^ is conserved in RABV strains.

We also explored the effect of amino acid mutations on the antigenicity of the M protein. We found that the D28G mutant but not the Y27D mutant eliminated the reactivity of mAbs. Single P26L or Y27D mutations within the epitope could not affect the binding of mAbs. This is the first record of precise mutations resulting in antigenic difference of the RABV M protein. This epitope is located at the N-terminus (17–70 amino acids), which is an antigenic region identified in a previous study [28].

To investigate the spectrum of detection, the mAbs developed here were used to detect RABV in the brains of infected mice using IHC. We found that mAbs could detect the virus in brain tissues of RABV-infected mice. We also found the mAbsreact against the CVS-11 but not the HEP-Flury strain. These findings suggest that mAbs can be used in the detection of different RABV in IHC.

In conclusion, we successfully developed five mAbs showing specificity and sensitivity to RABV, which were successfully used in IFA, WB, and IHC for RABV detection. Moreover, we identified a variable epitope (PPYDDD) making this study the first to report precise M protein epitope. Our study has potential applications in the diagnosis of RABV infection as well as the investigation of the motifs structure, function, and antigenicity of the RABV M protein. The role of the variable M protein epitope in virulence and pathogenicity of RABV still needs to be explored.

## Figures and Tables

**Table 1:** Primers for amplification of the overlapping and first round truncated segments of the M gene of rabies virus (RABV) CVS-11 strain.

Fragment	Primer Sequence (5′–3′)
1–203 aa	*GGGGTACC*ATGAACGTTCTACGCAAGAT (*Hin*dIII)
	*CCCAAGCTT*TTATTCTAGAAGCAGAGAAGAG (*Kp*nI)
1–107 aa	*GGGGTACC*ATGAACGTTCTACGCAAGAT (*Hin*dIII)
	*CCCAAGCTT*AGGTACTGGAGCTCCTGATAAA (*Kp*nI)
101–203 aa	*GGGGTACC*TTATCAGGAGCTCCAGTACCT (*Hin*dIII)
	*CCCAAGCTT*TTATTCTAGAAGCAGAGAAGAG (*Kp*nI)
1–32 aa	*GGGGTACC*ATGAACGTTCTACGCAAGATA (*Hin*dIII)
	*CCCAAGCTT*CCACAGGTCATCGTCATACGG (*Kp*nI)
26–36 aa	*GGGGTACC*TATGACGATGACCTGTGGCTTCCACCTCCT (*Hin*dIII)
	*CCCAAGCTT*AGGAGGTGGAAGCCACAGGTCATCGTCATACGG (*Kp*nI)
33–203 aa	*GGGGTACC*TGACCTGTGGCTTCCACCTC (*Hin*dIII)
	*CCCAAGCTT*TTATTCTAGAAGCAGAGAAGAG (*Kp*nI)
1–17 aa	*GGGGTACC*ATGAACGTTCTACGCAAGAT (*Hin*dIII)
	TAGGGATGAGGACACTCAAAAGCTTGGG (*Kp*nI)
17–26 aa	*GGGGTACC*CAAAAGCCCTCTCCTGTGT (*Hin*dIII)
	*CCCAAGCTT*CCACAGGTCATCGTCATACGG (*Kp*nI)

**Table 2:** Oligonucleotides coding for the second and third round truncated M protein of the epitopes.

Fragment	Primer Sequence (5′–3′)
17–25 aa	CCAAAAGCCCTCTCCTGTGTCAGCCCCTA
	CATGGGTTTTCGGGAGAGGACACAGTCGGGGATTCGA
24–32 aa	CGCCCCTCCGTATGACGATGACCTGTGGA
	CATGGCGGGGAGGCATACTGCTACTGGACACCTTCGA
24–31 aa	GATCCGCCCCTCCGTATGACGATGACCTGC
	TCGAGCAGGTCATCGTCATACGGAGGGGCG
24–30 aa	GATCCGCCCCTCCGTATGACGATGACC
	TCGAGGTCATCGTCATACGGAGGGGCG
24–29 aa	GATCCGCCCCTCCGTATGACGATC
	TCGAGATCGTCATACGGAGGGGCG
24–28 aa	GATCCGCCCCTCCGTATGACC
	TCGAGGTCATACGGAGGGGCG
25–32 aa	GATCCCCTCCGTATGACGATGACCTGTGGC
	TCGAGCCACAGGTCATCGTCATACGGAGGG
26–32 aa	GATCCCCGTATGACGATGACCTGTGGC
	TCGAGCCACAGGTCATCGTCATACGGG
27–32 aa	GATCCTATGACGATGACCTGTGGC
	TCGAGCCACAGGTCATCGTCATAG
28–32 aa	GATCCGACGATGACCTGTGGC
25–30 aa	TCGAGCCACAGGTCATCGTCG GATCCCCTCCGTATGACGATGACC TCGAGGTCATCGTCATACGGAGGG
25–30 aa (pCMV-Flag)	AGCTTCCTCCGTATGACGATGACGGTAC AGTCATCGTCATACGGAGGC

**Table 3:** Oligonucleotides coding for the mutant epitope fragment.

Fragment	Primer Sequence (5′–3′)
P26L mutation	GATCCCCTCTGTATGACGATGACC
	TCGAGGTCATCGTCATACAGAGGG
Y27D mutation	GATCCCCTCCGGATGACGATGACC
	TCGAGGTCATCGTCATCCGGAGGG
D28G mutation	GATCCCCTCCGTATGGCGATGACC
	TCGAGGTCATCGCCATACGGAGGG
